# The eEF2 kinase-induced STAT3 inactivation inhibits lung cancer cell proliferation by phosphorylation of PKM2

**DOI:** 10.1186/s12964-020-0528-y

**Published:** 2020-02-13

**Authors:** Min Xiao, Jianling Xie, Yu Wu, Genzhu Wang, Xin Qi, Zailiang Liu, Yuying Wang, Xuemin Wang, Ashfaqul Hoque, Jon Oakhill, Christopher G. Proud, Jing Li

**Affiliations:** 1 Key Laboratory of Marine Drugs, Chinese Ministry of Education, School of Medicine and Pharmacy, Ocean University of China, Qingdao, 266003 People’s Republic of China; 2 South Australian Health & Medical Research Institute, North Terrace, Adelaide, SA 5000 Australia; 3 School of Biological Sciences, University of Adelaide, Adelaide, SA 5005 Australia; 4 St Vincent’s Institute of Medical Research, Fitzroy, SA 4312 Australia; 5 Laboratory for Marine Drugs and Bioproducts of Qingdao National Laboratory for Marine Science and Technology, Qingdao, 266237 People’s Republic of China; 6 Open Studio for Druggability Research of Marine Natural Products, Pilot National Laboratory for Marine Science and Technology (Qingdao), Qingdao, People’s Republic of China

**Keywords:** eEF2K, PKM2, Dimerization, Proliferation, Cancer metabolism

## Abstract

**Background:**

Eukaryotic elongation factor-2 kinase (eEF2K) is a Ca 2+ /calmodulin (CaM)-dependent protein kinase that inhibits protein synthesis. However, the role of eEF2K in cancer development was reported paradoxically and remains to be elucidated.

**Methods:**

Herein, A549 cells with eEF2K depletion or overexpression by stably transfected lentivirus plasmids were used in vitro and in vivo study. MTT and colony assays were used to detect cell proliferation and growth. Extracellular glucose and lactate concentration were measured using test kit. Immunoblot and co-immunoprecipitation assays were used to examine the molecular biology changes and molecular interaction in these cells. LC-MS/MS analysis and [γ- 32 P] ATP kinase assay were used to identify combining protein and phosphorylation site. Nude mice was utilized to study the correlation of eEF2K and tumor growth in vivo.

**Results:**

We demonstrated that eEF2K inhibited lung cancer cells proliferation and affected the inhibitory effects of EGFR inhibitor gefitinib. Mechanistically, we showed that eEF2K formed a complex with PKM2 and STAT3, thereby phosphorylated PKM2 at T129, leading to reduced dimerization of PKM2. Subsequently, PKM2 impeded STAT3 phosphorylation and STAT3-dependent c-Myc expression. eEF2K depletion promoted the nuclear translocation of PKM2 and increased aerobic glycolysis reflected by increased lactate secretion and glucose.

**Conclusions:**

Our findings define a novel mechanism underlying the regulation of cancer cell proliferation by eEF2K independent of its role in protein synthesis, disclosing the diverse roles of eEF2K in cell biology, which lays foundation for the development of new anticancer therapeutic strategies.

## Background

Eukaryotic elongation factor-2 kinase (eEF2K) is a Ca^2+^/calmodulin (CaM)-dependent protein kinase that inhibits the elongation stage of protein synthesis [1, 2]. eEF2K phosphorylates the known substrate, translation elongation factor eEF2, on Thr56, resulting in inhibition of eEF2 activity [3, 4] and translocation process of elongation, during which peptidyl-tRNAs is moved from the A- to the P-site of ribosome and ribosome is moved by one codon along the mRNA [5]. A high proportion of cellular energy and amino acids in rapidly-dividing cells are consumed in protein synthesis, and almost all (> 99%) of this consumption occurs in the elongation stage [6].

Oncogenic transformation in tumorigenesis is accompanied by increased dependence on nutrients, particularly glucose and glutamine, thus rendering the cells particularly sensitive to inadequate nutrient supply [7]. Cancer cells within the tumor microenvironment often encounter energy stress including nutrient deprivation and hypoxia due to insufficient tumor vascularization [7, 8]. To balance tumorigenic events that drive energy-demanding processes, such as proliferation, cancer cells possess or invoke adaptive cytoprotective responses [9]. eEF2K is an evolutionarily-conserved mediator of cellular responses to nutrient deprivation [10] and is expressed at high levels in certain cancers [11]. Its activity is controlled by nutrient-regulated signaling pathways, activated by AMP-activated protein kinase (AMPK) under nutrient deprivation, and inhibited by the mTORC1 [12–15]. In contrast to the findings that support the tumor-promoting role of eEF2K, two studies showed that eEF2K impedes the initiation and growth of intestinal tumors [16, 17], suggesting that the role of eEF2K in cancer is more complex than simply exerting a cytoprotective effect.

Pyruvate kinase M2 isoform (PKM2) functions as a rate-limiting glycolytic enzyme that catalyzes conversion of PEP to pyruvate and transfers a phosphate from PEP to ADP [18]. Warburg effect or aerobic glycolysis is a prominent metabolic feature of cancer cells, and PKM2 plays a key role in such metabolic reshuffle [19, 20]. Recently years, PKM2 has been extensively studied in non-glycolytic activity and exhibits various biological functions in tumor progression [21–23], for example, nuclear dimer PKM2 as a protein tyrosine kinase directly phosphorylates the signal transducer and activator of transcription 3 (STAT3) at T705, thereby contributing to drive cancer cell proliferation [24]. Moreover, PKM2 in tumor cells mediates resistance acquisition to various chemotherapeutic drugs, such as gefitinib [25, 26].

In this study, we unexpectedly found that eEF2K acts as cancer suppressor distinct from its role of cytoprotection in lung cancer cells. We demonstrate that eEF2K blocks conversion of tetramer to dimer of PKM2 by phosphorylating PKM2 at Thr129, leading to inhibition of PKM2-mediated activation of STAT3 and Myc. Thereby, eEF2K inhibits the aerobic glycolysis of lung cancer cells and then impairs cells proliferation and tumor growth. These effects of eEF2K are independent of its role in regulating protein synthesis.

## Methods

### Cell culture and reagents

The human lung cancer A549 cells were purchased from Shanghai Cell Bank, Chinese Academy of Science. A549 cells, H1299 cells and A549 cells containing either non-targeting control or eEF2K-targeted shRNA (referred to as A549 shNC and A549 sheEF2K; or referred to as siNC and sieEF2K) were cultured in F12K medium. HEK293 cells used for virus packaging were maintained in Dulbecco’s modified Eagle medium (DMEM). All mediums were supplemented with 10% FBS, 100 units/ml penicillin and 0.1 mg/ml streptomycin. Cells were maintained at 37 °C in a humidified cell incubator containing 5% CO_2_ and 95% air.

A484954, BS^3^, Gefitinib and Stattic were purchased from Sigma-Aldrich. Antibodies For EGFR (#2646), p-EGFR (#3777), ERK (#4695), p-ERK (#4370), PDGFR (#3174), p-PDGFR (#3124), JAK1 (#3344), p-JAK1 (#74129), JAK2 (#3230), p-JAK2 (#3771), p-PI3K (#4228), AKT (#4685), p-AKT (#9614), Src (#12109), p-Src (#12432), STAT3 (#12640), p-STAT3 (#9145), eEF2K (#3692), eEF2 (#2332), p-eEF2 (#2331), c-Myc(#2729), and PKM2 (#4053) were obtained from Cell Signaling Technology. β-Actin, Tubulin, goat anti-rabbit and goat anti-mouse secondary antibodies were all purchased from Hangzhou HuaAn Biotechnology Co. (PCNA antibody(#101118-T46) was purchased from Sino Biolgical. Protein A/G plus agarose was purchased from Santa Cruz Biotechnology.

### Lentiviral infection

A549 cells in exponential phase of growth were plated into 24-well tissue culture plates at 8.0 × 10^4^ cells per well, then infected with either eEF2K-targeted shRNA lentiviral particles (Santa Cruz, sc-39,011-V) or control shRNA lentiviral particles (Santa Cruz, sc-108,080) for 24 h, stable expression clones were screened with antibiotic puromycin for 14 days.

### Transfection with siRNA and DNA plasmids

Small interfering RNAs (siRNAs) were purchased from GenePharma. Human eEF2K gene silence was performed using an siRNA duplex targeting the following sequence (referred as SieEF2K): sense 5′-GUCAAUUCCAAGGUUAAUATT-3′ and antisense 5′-UAUUAACCUGGAAUUGACTT-3′; Human STAT3 was silenced using siRNA duplexes targeting the following sequences: sense 5′-GCAACAGAUUGCCUGCAUUTT-3′ and antisense 5′-AAUGCAGGCAAUCUGUUGCTT-3′; Human PKM2 was targeted with siRNA duplexes targeting the following sequences: sense 5′-GAAUGAAUGUGGCUCGUCUdTdT-3′ and antisense 5′-AGACGAGCCACAUUCAUUCdTdT-3′; A non-targeting siRNA was used as a control (referred as SiNC): sense 5′-UUCUCCGAACGUGUCACGUTT-3′ and antisense 5′-ACGUGACACGUUCGGAGAATT-3′. In brief, A549 cells in exponential phase of growth were plated into six-well tissue culture plates at 1 × 10^5^ cells per well, transfected with each duplex siRNA using Lipofectamine 3000 according to the manufacturer’s protocol. Flag-eEF2K WT plasmid was described and performed as the previous report (referred as WT-eEF2K) [11].

### Cell proliferation assays

A549 cells, A549 shNC and A549 sheEF2K stable cells were cultured for the indicated times. For instantly transfected cells, A549 cells were plated at 1.5 × 10^5^ cells per well into 6-well tissue culture plates and incubated at 37 °C in a humidified atmosphere containing 5% CO_2_ for 12 h, then transfected with eEF2K siRNAs or WT plasmid and cultured for the indicated times with or without Gefitinib. For serum or glucose deprivation, A549 cells, A549 shNC and A549 sheEF2K stable cells were cultured with serum-free or glucose-free medium for the indicated times. Cell proliferation was assessed by MTT assay. Cells were incubated with MTT for 4 h at 37 °C, and 150 μl DMSO was added, then the number of proliferation cells was determined by measuring the intensity of absorption at 490 nm. Cell proliferation = (Control group-treated group)/Control group × 100%.

### Colony assays

A549 cells, A549 shNC and A549 sheEF2K cells were seeded at a density of 500 cells per well into 6-well plates and cultured for 15 days. The cells were fixed with anhydrous methanol and stained with Giemsa, and the number of colonies was counted under the microscope.

### Extracellular lactate and glucose measurement

Cells were seeded into 6-well plates, after 12 h the culture media were replaced with fresh complete medium and incubated for additional 6 h. The media were then collected for extracellular measurement of glucose and lactate concentration. Cells were harvested for protein lysates including for normalization of cell number. Lactic acid levels were determined by using a lactic acid test kit (KeyGEN Biotech, KGT023) according to the manufacturer’s instruction. Glucose levels were measured using a glucose (GO) assay kit (Sigma-Aldrich, GAGO-20). All values were normalized on the basis of the Bradford protein assay.

### SDS-PAGE/Western blot analysis and co-immunoprecipitation

Cells were lysed in cell lysis buffer for Western & IP (Beyotime Biotechnology) supplemented with a protease inhibitor PMSF at 4 °C for 30 min. Xenografted tumour tissues were homogenized in cell lysis buffer for Western. Protein concentrations of the lysates were measured using the BCA reagent (Beyotime Biotechnology). Equal amounts of protein were run on 10% SDS-PAGE gels, then transferred to nitrocellulose membranes (Pall), and probed with the indicated primary antibodies. Anti-rabbit and anti-mouse (1:5000) HRP-conjugated secondary antibodies were used. Blots were detected by chemiluminescence with the enhanced chemiluminescence detection reagents (PIERCE). For co-immunoprecipitation, cell lysates were incubated overnight at 4 °C with eEF2K (Santa Cruz, sc-393,366) or PKM2 (Santa Cruz, sc-365,684) antibody, then conjugated to protein A/G agarose beads while rocking. Immunoprecipitates were washed with washing buffer (50 mM Tris (pH 7.5), 150 mM NaCl, 1% Triton-X), re-suspended in 2 × loading buffer, and resolved by SDS-PAGE followed by immunoblotting analysis. The immunoprecipitates with eEF2K antibody were also subjected to protein MS analysis. Blots were quantified using Image J software and expressed by graphs.

### eEF2K activity and phosphorylation level of PKM2 assays

Recombinant GST-tagged eEF2K prepared in *E. coli* as previously described [27]. GST-tagged eEF2K, recombinant PKM2 (Abcam, ab89364) or eEF2 prepared in *E. coli* as previously described [28] were incubated in eEF2K kinase assay buffer (2 mM EDTA, 0.4 mM EGTA, 0.67 mM CaCl_2_, 5 mM MgCl_2_, 50 mM MOPS pH 7.0) containing 40 μg/ml or 16 ng/assay CaM (unless where specified), 50 μM unlabelled ATP, 1 μCi [γ-32P]ATP and where specified, 5 μM JAN-384 at 30 °C for the indicated periods of times. Samples were taken at 5, 10, 15, and 30 min by spotting 8-μl aliquots from the 40-μl assay mixture onto squares of Whatman P81 paper (2 cm by 2 cm), which were washed three times (5 min each) in 75 mM phosphoric acid followed by methanol before drying in air and scintillation counting.

### In-gel trypsin digestion and LC-MS/MS analysis

Following in-vitro eEF2K kinase assay and SDS-PAGE run, PKM2 bands were excised from the gel. Excised bands were destained with 50 mM Triethyl Ammonium Bicarbonate (TEAB) (50%)/acetonitrile (50%) overnight and subsequently 30 min on a rotation device. Gel plugs were dehydrated for 30 min with 100% acetonitrile. Dehydrated gel plugs were reduced with 10 mM Tris (2-Carboxyethyl) phosphine (TCEP) for 45 min at 55 °C and alkylated with 55 mM iodoacetamide at room temperature in the dark for 30 min. Gel pieces were washed 3 times with 50 mM TEAB for 10 min each on a rotation device before they were dehydrated with 100% acetonitrile. Dehydrated gel plugs were digested with trypsin (Sigma# T7575) dissolved in 25 mM TEAB at 37 °C overnight. Digested tryptic peptides were freeze-dried and resuspended in 0.1% (v/v) formic acid and analyzed by LC-MS/MS using a Q-Exactive plus mass spectrometer (Thermo Scientific) fitted with nanoflow reversed-phase-HPLC (Ultimate 3000 RSLC, Dionex). The nano-LC system was equipped with an Acclaim Pepmap nano-trap column (Dionex-C18, 100 Å, 75 μm × 2 cm) and an Acclaim Pepmap RSLC analytical column (Dionex-C18, 100 Å, 75 μm × 50 cm). Typically for each LC-MS/MS experiment, 5 μL of the peptide mix was loaded onto the enrichment (trap) column at an isocratic flow of 5 μL/min of 3% (v/v) acetonitrile containing 0.1% (v/v) formic acid for 6 min before the enrichment column is switched in-line with the analytical column. The eluents used for the LC were 0.1% (v/v) formic acid (solvent A) and 100% acetonitrile/0.1% formic acid (v/v) (solvent B). The gradient used was 3% B to 25% B for 23 min, 25% B to 40% B in 2 min, 40% B to 80% B in 2 min and maintained at 85% B for the final 2 min before equilibration for 9 min at 3% B prior to the next analysis. All spectra were acquired in positive mode with full scan MS spectra scanning from m/z 375–1400 at 70000 resolution with AGC target of 3e6 with maximum accumulation time of 50 ms. Lockmass of 445.120024 was used. The 15 most intense peptide ions with charge states ≥2–5 were isolated with isolation window of 1.2 m/z and fragmented with normalized collision energy of 30 at 35000 resolution with AGC target of 1e5 with maximum accumulation time of 120 ms. Underfill threshold was set to 2% for triggering of precursor for MS2. Dynamic exclusion was activated for 30s. Mass spectrometric raw data were searched using Mascot search algorithm against human SwissProt database. Cysteine carbamidomethylation was searched as a fixed modification, whereas oxidation of methionine, phosphorylation of serine, threonine and tyrosine were searched as variable modifications.

### Vectors and mutagenesis

pRK7-FLAG-Rheb was described in previously published paper [28]. pWZL-FLAG-PKM2 was purchased from Addgene (#20585). Point mutations were introduced by PCR mutagenesis using the Pfu DNA polymerase (Promega) using the following primers (all for human PKM2): T41A: Forward: 5′ CACCACCCATCGCAGCCCGGAAC 3′ and Reverse: 5′ GTTCCGGGCTGCGATGGGTGGTG 3′. S55A: Forward: 5′ CATTGGCCCAGCTGCCCGATCAGTGG 3′ and Reverse: 5′ CCACTGATCGGGCAGCTGGGCCAATG 3′. T60A: Forward: 5′ CCGATCAGTGGAGGCGTTGAAGGAGATG 3′ and Reverse: 5′ CATCTCCTTCAACGCCTCCACTGATCGG 3′. T87A: Forward: 5′ GTACCATGCGGAGGCCATCAAGAATGTG 3′ and Reverse: 5′ CACATTCTTGATGGCCTCCGCATGGTAC 3′. T129A: Forward: 5′ AAGGGCAGCGGCGCTGCAGAGGTGGA 3′ and Reverse: 5′ TCCACCTCTGCAGCGCCGCTGCCCTT 3′. S249A: Forward: 5′ ATCCGCAAGGCAGCCGATGTCCATGAAG 3′ and Reverse: 5′ CTTCATGGACATCGGCTGCCTTGCGGAT 3′. S333A: Forward: 5′ GATGCTGGAGGCCATGATCAAG 3′ and Reverse: 5′ CTTGATCATGGCCTCCAGCATC 3′. T412A: Forward: 5′ CCACAGAAGCCGCCGCCGTGGG 3′ and Reverse: 5′ CCCACGGCGGCGGCTTCTGTGG 3′. HEK293 cells were maintained in Dulbecco’s modified Eagle medium (DMEM) media containing 10% (v/v) FBS and 1% penicillin/streptomycin. Cells were cultured at 37 °C in 5% CO2 and 95% air. HEK293 cells were transfected by the calcium phosphate method as previously described [29].

### PKM2 dimerization and tetramerization measurement

Cells were washed twice with PBS (pH 8.0) before treated with BS^3^ for 1 h at room temperature. And then lysed in 2 × loading buffer (0.125 M Tris (pH 6.8), 3% SDS, 10% glycerin, 0.02% BPB, and 5% β-mercaptoethanol) at 4 °C for 30 min. Levels of PKM2 dimerization and tetramerization were analyzed by immunoblotting.

### Mouse studies

Nude mice were inoculated subcutaneously with A549 shNC and A549 sheEF2K stable cells (8 × 10^6^ cells/per site). Cancer growth was periodically monitored after post-injection for 40 days. Cancer volume was estimated using the following formula: cancer length × (cancer width)^2^ × 0.5. Half of the dissected cancer issues were fixed with formalin for subsequent immunohistochemistry (IHC) analysis to detect PCNA (proliferating cell nuclear antigen) expression, and the other half were lysed on ice, followed by immunoblotting analysis of indicated proteins.

### Statistical analysis

Data in graphs are presented as mean ± sd. Statistical significance was analyzed by one-way ANOVA for at least three independent experiments, and *P* < 0.05 was considered significant.

## Results

### eEF2K inhibits lung cancer cell proliferation and affects the effects of gefitinib

**Fig. 1:**
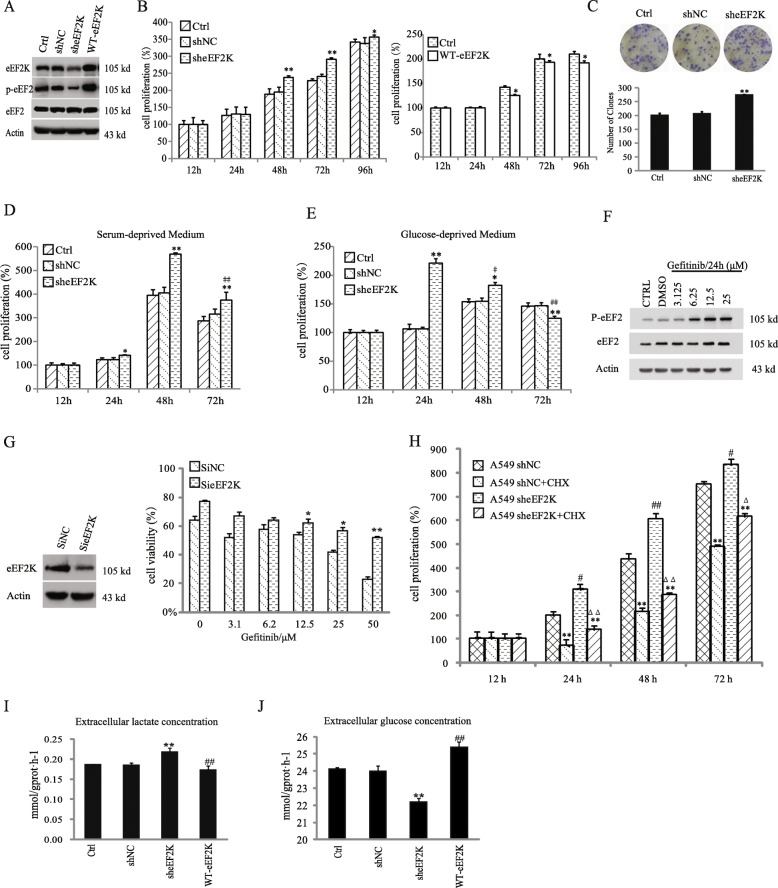
eEF2K inhibition promotes lung cancer cell proliferation of and alleviates the effects of gefitinib. **a** A549 cells were transfected with the indicated plasmids. Immunoblotting analyses were performed with the indicated antibodies. **b** A549 cells with or without eEF2K depletion or overexpression were cultured for the indicated periods of time. MTT assay was performed. *n* = 5. *P < 0.05, **p < 0.01, vs shNC or Ctrl. **c** A549 cells with or without eEF2K depletion were cultured for 15 days, fixed with methanol, and then stained with Giemsa. The clone numbers were counted under microscope. *n* = 3. **p < 0.01, vs shNC. **d**, **e** A549 cells with or without eEF2K depletion were cultured in glucose-deprived medium (**d**) or serum-deprived medium (**e**). The cell proliferation was examined by MTT assay. *n* = 5. *P < 0.05, **p < 0.01, vs shNC. ^##^*p* < 0.01, vs sheEF2K (48 h or 24 h). **f** A549 cells were treated with Gefitinib at the indicated concentrations. Immunoblotting analyses were performed with the indicated antibodies. **g** A549 expressing with or without eEF2K siRNA were treated with Gefitinib at the indicated concentrations. Immunoblotting analyses were performed with the indicated antibodies. The cell viabilities were determined by MTT assay, The histogram shows the percentages of the viable cells. *P < 0.05, **p < 0.01, vs SiNC. (H) A549 cells with or without eEF2K depletion were treated with or without CHX. The cell proliferation were determined by MTT assay. *P < 0.05, **p < 0.01, vs no treated CHX. ^#^*P* < 0.05,^##^*P* < 0.01, vs shNC. ^ΔΔ^*P* < 0.01, vs shNC+CHX. **i**-**j** A549 cells with or without eEF2K depletion or overexpression were cultured in serum-free medium for 12 h followed by complete medium for 12 h. The culture medium and cell lysate were examined for lactate secretion (**i**), glucose amount in medium (**j**). **P < 0.01, vs shNC. ^#^*P* < 0.05, ^##^*P* < 0.01, vs Ctrl

To investigate the role of eEF2K in cancer cell proliferation, we depleted or overexpressed eEF2K in A549 non-small lung cancer cells (Fig. 1a). Expression of eEF2K shRNA significantly increased A549 cell proliferation whereas eEF2K overexpression largely decreased the cell proliferation, as detected by MTT assay (Fig. 1b). We further examined cell growth using plate colony formation assays and found that eEF2K depletion promoted cell growth (Fig. 1c), further supporting that eEF2K inhibits lung cancer cell proliferation.

We next determined the role of eEF2K in cell proliferation under condition of nutrient deprivation. As is reported [10], serum and glucose deprivation decreased eEF2K phosphorylation at Ser366 and Ser78, but increased eEF2K phosphorylation at Thr56 in a time-dependent manner (Additional file 1: Figure S1). Intriguingly, eEF2K-depleted cells with deprived glucose (Fig. 1d) or serum (Fig. 1e) initially proliferated faster than their eEF2K-undepleted cells and then underwent more growth inhibition at the time of nutrient consumption, suggesting that eEF2K inhibits lung cancer cell proliferation other than mediating cytoprotection under condition of nutrient deprivation in A549 cells.

It was also reported that eEF2K has cytoprotection effects against chemotherapies besides nutrient deprivation [30, 31]. We treated A549 cells with EGFR inhibitor gefitinib and found that gefitinib treatment induced eEF2 Thr56 phosphorylation in a dosage-dependent manner (Fig. 1f) and reduced cell viability, and this inhibitory effect was alleviated by eEF2K depletion (Fig. 1g) or treatment of the cells with eEF2K inhibitor A484954 [31] (Additional file 1: Figure S2). These results strongly suggested that gefitinib treatment promotes lung cancer cell death through eEF2K activation, consolidating the notion that in A549 cells, eEF2K inhibits cell proliferation.

To determine whether eEF2K inhibition-enhanced cell proliferation is due to abrogation of the inhibitory effect of eEF2K on protein synthesis, we treated A549 cells with protein synthesis inhibitor cycloheximide (CHX). Figure 1h showed that CHX depletion failed to block eEF2K depletion-enhanced cell proliferation, suggesting that eEF2K inhibition-promoted on cell proliferation is not due to its effect on protein translation elongation. Interestingly, eEF2K depletion significantly increased lactate production and glucose utilization, and overexpression of eEF2K led to a reduction in lactate secretion and glucose consumption (Fig. 1i-j), which was consistent with the positively regulation in the glycolytic enzyme activity of PKM2 by eEF2K (Additional file 1: Figure S3). These results suggested that eEF2K has the ability to regulate glycolysis in these cancer cells.

### eEF2K inhibition increases STAT3 phosphorylation

**Fig. 2:**
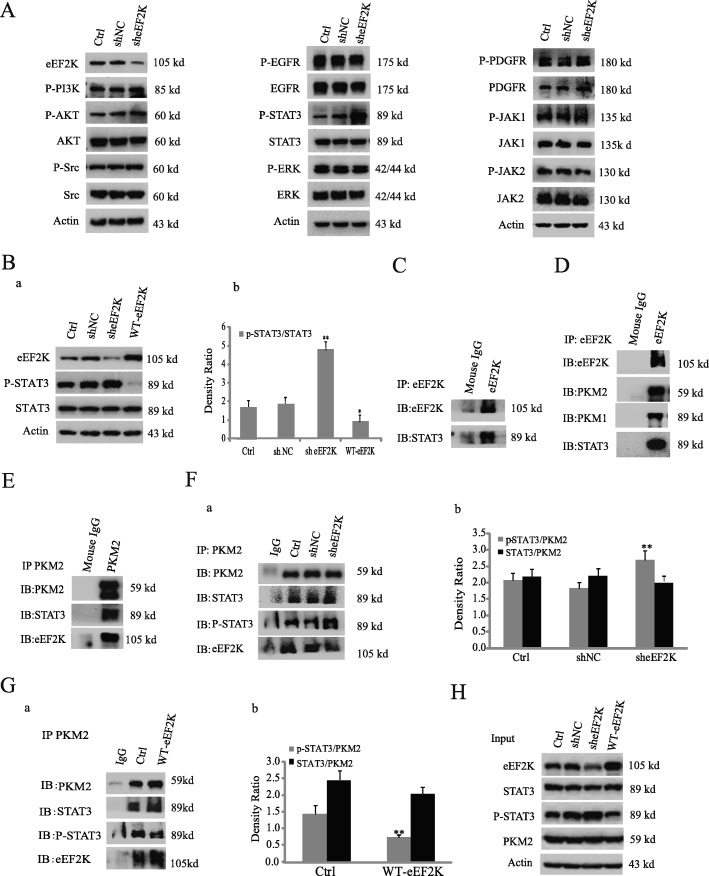
eEF2K inhibition increases STAT3 phosphorylation. (**A**) A549 cells with or without eEF2K depletion were analyzed by immunoblotting analyses with the indicated antibodies. (**B**) A549 cells with or without eEF2K depletion or overexpression were analyzed by immunoblotting analyses with the indicated antibodies (Ba). The relative phosphorylation levels of the STAT3 to STAT3 were quantified (Bb). (**C**) eEF2K was immunoprecipitated from lysates of A549 cells. Immunoblotting analyses were performed with the indicated antibodies. (**D**, **E**) A549 cell lysates were prepared. Immunoprecipitation and immunoblotting analyses were performed with the indicated antibodies. (**F**, **G**) Lysates of A549 cells with or without eEF2K depletion (Fa) or overexpression (Ga) were immunoprecipitated with an anti-PKM2 antibody. Immunoblotting analyses were performed with the indicated antibodies. The relative phosphorylation levels of the STAT3 to PKM2 were quantified (Fb and Gb). **P < 0.01, vs shNC or **P < 0.01, vs Crtl. (**H**) Lysates of A549 cells with or without eEF2K depletion or overexpression were analyzed by immunoblotting analyses with the indicated antibodies

To explore the mechanism underlying eEF2K-mediated cell proliferation, we examined major signaling pathways related to cell proliferation. We found that eEF2K depletion prominently increased STAT3 phosphorylation, but not the phosphorylation and expression of EGFR, PDGFR, PI-3 K, Src, AKT, JAK1, JAK2, and ERK (Fig. 2A). Conversely, eEF2K overexpression obviously reduced the STAT3 phosphorylation (Fig. 2Ba, Bb). Similar results were also observed in H1299 non-small lung cancer cells (Additional file 1: Figure S4). Co-immunoprecipitation (Co-IP) analyses by eEF2K antibody showed that eEF2K interacted with STAT3 (Fig. 2C), and Mass spectrometry (MALDI-TOF/MS/MS) analysis of the eEF2K immunoprecipitates revealed that PKM was an associated protein with eef2k in a high percentage sequence coverage (80.4%). PKM is a glycolytic enzyme that catalyzes the transfer of a phosphoryl group from phosphoenolpyruvate (PEP) to ADP, generating ATP [18]. The M1 and M2 forms are produced from the PKM gene by differential splicing [32]. Further immunobloting analyses of eEF2K immunoprecipitates showed that PKM1, PKM2, and STAT3 were associated with eEF2K (Fig. 2D). Reciprocal IP with an anti-PKM2 antibody confirmed that eEF2K and STAT3 were associated with PKM2 (Fig. 2E). These results indicated that that eEF2K, PKM2 and STAT3 are in a protein complex in A549 cells.

Given that PKM2, but not PKM1, phosphorylates STAT3 [24], we next investigate whether PKM2 plays a role in eEF2K-mediated STAT3 phosphorylation. Depletion or overexpression of eEF2K had no effect on the PKM2-STAT3 interaction in A549 cells, but increased or decreased PKM2-associated phosphorylation level of STAT3 in eEF2K-depleted (Fig. 2Fa, Fb) or overexpressed (Fig. 2Ga, Gb) A549 cells, respectively. These results suggested that eEF2K regulates STAT3 phosphorylation through PKM2.

**Fig. 3:**
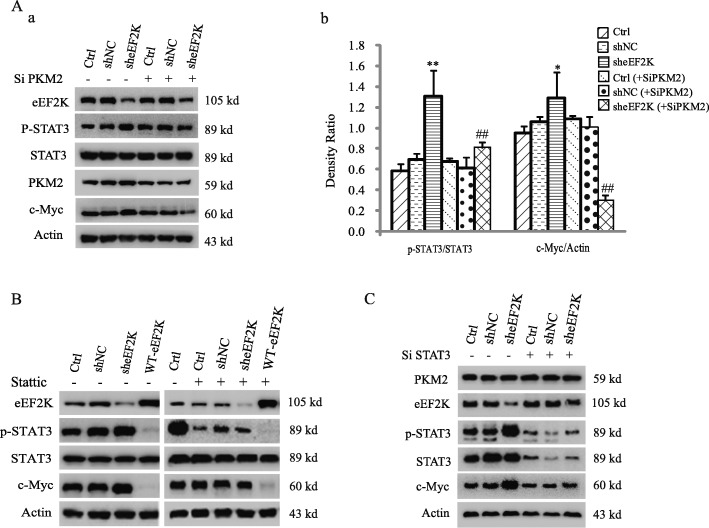
eEF2K regulates c-Myc expression in a PKM2- and STAT3-depdendent manner. (**A**) A549 cells with or without eEF2K depletion were transfected with or without PKM2 depletion. Immunoblotting analyses were performed with the indicated antibodies (Aa). The relative expression levels of the indicated proteins were quantified (Ab). *P < 0.01, **P < 0.01, vs shNC. ^##^*P* < 0.01, vs sheEF2K. (**B**) A549 cells with or without eEF2K depletion or overexpression were treated with or without stattic. Immunoblotting analyses were performed with the indicated antibodies. (**C**) A549 cells with or without eEF2K depletion were transfected with or without STAT3 siRNA. Immunoblotting analyses were performed with the indicated antibodies

To further explore the effects of PKM2 on the phosphorylation of STAT3, we depleted PKM2 by siRNA-mediated knockdown and found that the increase of p-STAT3 due to knockdown of eEF2K was distinctly reversed compared to cells treated with siPKM control. Notably, the expression of c-Myc, which regulates aerobic glycolysis and promotes tumor progression in various cancer [33], were correlated with phosphorylation levels of STAT3 (Fig. 3Aa and Ab), and overexpression of eEF2K markedly decreased STAT3 phosphorylation, but not total STAT3 expression, with corresponding reduction of c-Myc expression (Fig. 3B). To determine whether STAT3 regulates c-Myc expression in response to alteration of eEF2K, we treated A549 cells with stattic, a specific and irreversible STAT3 inhibitor [34]. As shown in Fig. 3B, stattic inhibited eEF2K depletion-enhanced STAT3 phosphorylation and c-Myc expression. Similar results were obtained by expression of siRNA of STAT3 (Fig. 3C). Altogether, our findings support that eEF2K inhibits PKM2-depdendent phosphorylation of STAT3 and subsequent c-Myc expression.

### eEF2K inhibits PKM2 dimerization

**Fig. 4:**
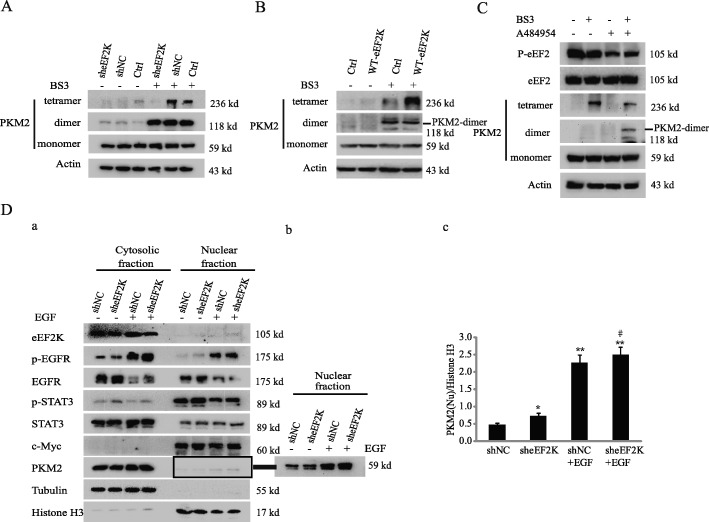
eEF2K inhibits PKM2 dimerization. (**A, B**) A549 cells with or without eEF2K depletion (**A**) or overexpression (**B**) were treated with BS^3^ for 1 h before being lysed. Immunoblotting analyses were performed with the indicated antibodies. (**C**) A549 cells were cultured in serum-free medium for 12 h, then treated with A484954 for 6 h followed by BS^3^ treatment for 1 h. Immunoblotting analyses were performed with the indicated antibodies. (**D**) A549 cells with or without eEF2K depletion were treated with or without EGF for 6 h. The nuclear and cytoplasmic proteins were extracted. Immunoblotting analyses were performed with the indicated antibodies (a). The expressions of PKM2 in nuclear extracts were presented in more obvious manner (b) and the relative levels of the PKM2 to Histone H3 were quantified (c). **P < 0.01, vs no treated EGF. ^#^*P* < 0.05, vs shNC+EGF

PKM2 in dimer, which has lower activity than its tetramer in phosphorylating pyruvate, acts as a protein kinase and phosphorylates STAT3 at Y705 in the nucleus [24]. To explore the mechanism by which eEF2K mediates PKM2-dependent STAT3 phosphorylation, we examined the oligomerization of PKM2 in eEF2K-depleted A549 cells. As shown in Fig. 4A, treated with BS3, a protein crosslinker [35], for 1 h before cell lysis, eEF2K depletion obviously shifted oligomerization of PKM2 from tetramers to dimers. Conversely, overexpression of eEF2K increased PKM2 tetramerization with reduce of its dimerization (Fig. 4B). Similar results to eEF2K depletion were obtained by treating the cells with eEF2K inhibitor A484954 (Fig. 4C). It has been reported that EGF stimulation promotes the translocation of PKM2 into the nucleus and subsequently upregulates the expression of c-Myc, thereby promoting the Warburg effect [26]. In the presence of EGF, we also found that nuclear PKM2 was increased. Furthermore, increased PKM2 in the nucleus was more observed in eEF2K-depleted cells (Fig. 4Da, Dc). We also found that in the nucleus extracts, PMK2 promoted the phosphorylation of STAT3 in the presence of PEP. Overexpression of eEF2K significantly decreased the phosphorylation of STAT3, and this inhibitory effect was reversed by eEF2K depletion. These effects were not obviously in the cytoplasmic extracts (Additional file 1: Figure S5). These results support that eEF2K regulates the dimerization and nuclear translocation of PKM2.

### eEF2K phosphorylates PKM2 at Thr129

**Fig. 5:**
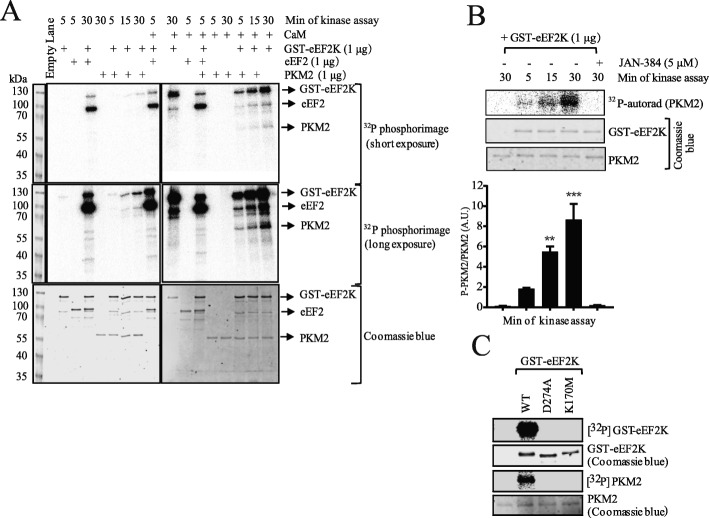
eEF2K phosphorylates PKM2. **a** An in vitro phosphorylation assay was performed with the indicated periods of time by incubation of 1 μg eEF2K, 8 μM ATP, and 1 μg of recombinant PKM2 or eEF2 in the presence or absence of CaM. Phosphorylation levels of substrates were detected by autoradiography. *: non-specific band. **b** An in vitro phosphorylation assay was performed with the indicated periods of time by incubation of 1 μg eEF2K, 8 μM ATP, CaM, and 1 μg of recombinant PKM2 in the presence or absence of 5 μM JAN-384 (upper panel). The relative phosphorylation levels of the PKM2 were quantified (lower panel). Data are presented as means ± S.E.M., *n* = 3, ** *P* < 0.01, ***P < 0.001. **c** An in vitro phosphorylation assay was performed by incubation of 8 μM ATP, CaM, and 1 μg of recombinant PKM2 in the presence of WT eEF2K, eEF2K D274A, or eEF2K K170M mutants

Given that eEF2K is a protein kinase and associates with PKM2, we examined eEF2K protein kinase activity by an in vitro eEF2K kinase assays using [γ-^32^P] ATP and eEF2 or PKM2 as substrates. As shown in Fig. 5a, eEF2K phosphorylated PKM2 in a time- and concentration-dependent manner, but not in the absence of CaM, an essential activator of eEF2K. This phosphorylation was inhibited by the eEF2K inhibitor JAN-384 [36] (Fig. 5b) or inclusion of eEF2K Kinase-dead (D274A and K170M) mutant, which was unable to autophosphorylate itself (Fig. 5c). As expected, eEF2K phosphorylated eEF2 (Additional file 1: Figure S6A). In addition, the extent of phosphorylation eEF2 by eEF2K was several times faster than that of PKM2, suggesting that eEF2 is still a better eEF2K substrate than PKM2 (Additional file 1: Figure S6B-C).

**Fig. 6:**
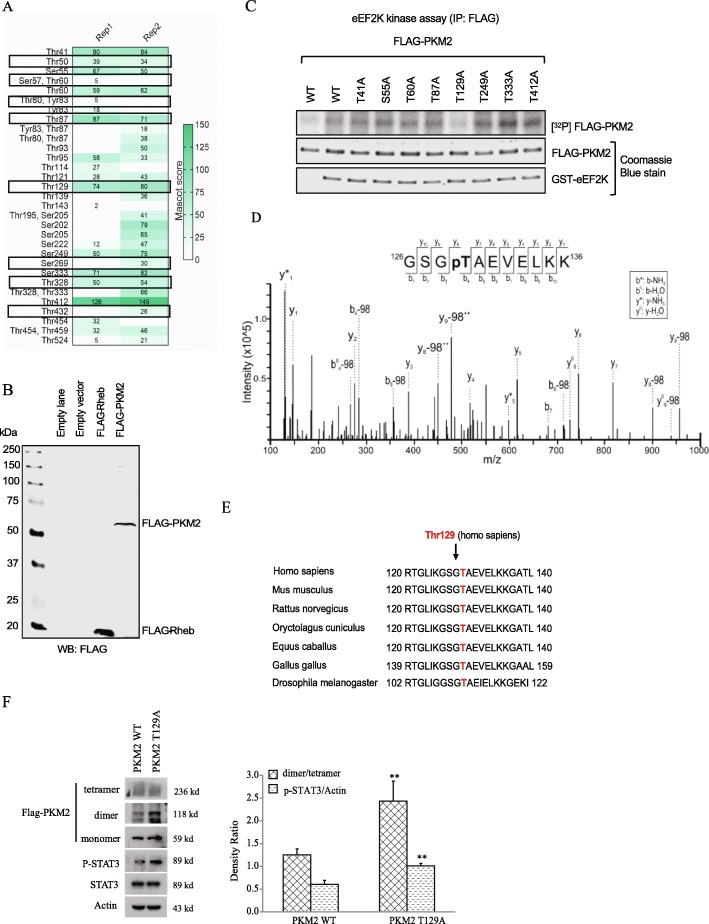
eEF2K phosphorylates PKM2 at Thr129. **a** Possible eEF2K phosphorylation sites in PKM2 identified by LC-MS/MS analysis following eEF2K kinase assays (2 replicates: Rep 1 and Rep 2) and in-gel digestion of PKM2. MS analysis. Selected potential phosphorylation sites are circled. **b** HEK293 cells were transfected with empty vector, FLAG-Rheb or FLAG-PKM2 constructs, cells were lysed 48 h after transfection and samples were immunoblotted using anti-FLAG. **c** WT FLAG-PKM2 and the indicated FLAG-PKM2 mutant proteins were expressed in HEK293 cells. FLAG-PKM2 was immunoprecipitated and then incubated with recombinant eEF2K kinase (1 μg) for 30 min for an in vitro phosphorylation assay. **d** Sequence alignment of the region of PKM2 Thr129 (in *Homo sapiens*) with other species. **e** MS-spectra shows PKM2 T129 phosphorylation. **f** A549 cells expressing WT PKM2 or PKM2 T129A were treated with BS^3^ for 1 h before being lysed. Immunoblotting analyses were performed with the indicated antibodies. The relative levels of the PKM2 and P-STAT3 to actin were quantified. **P < 0.01, vs. PKM2 WT.

To identify the phosphorylation residue of PKM2 by eEF2K, we performed in vitro phosphorylation and in-gel trypsin digestion of PKM2 for LC-MS/MS analysis and revealed 8 potential phosphorylation sites on PKM2 (Fig. 6a). Figure 6b shows that the FLAG-PKM2 in the assays is indeed FLAG-PKM2 (it is recognized by the FLAG Ab and at the right size). Mutation of these residues into alanine (A) showed that only FLAG-PKM2 T129A, which is highly conserved among different species, was resistant to eEF2K-mediated PKM2 phosphorylation (Fig. 6c-d), which is consistent with PKM2 T129 phosphorylation identified by LC-MS/MS analysis (Fig. 6e).

Of note, FLAG-PKM2 T129A exhibited increase of PKM2 dimerization amount accompanied by decrease of tetramerization in A549 cells compared to wild-type (WT) PKM2 and enhanced phosphorylation of STAT3 (Fig. 6f), These results suggested that eEF2K-mediated PKM2 T129 phosphorylation blocks conversion of tetramer to dimer of PKM2.

### eEF2K depletion promotes tumor growth in mice

**Fig. 7:**
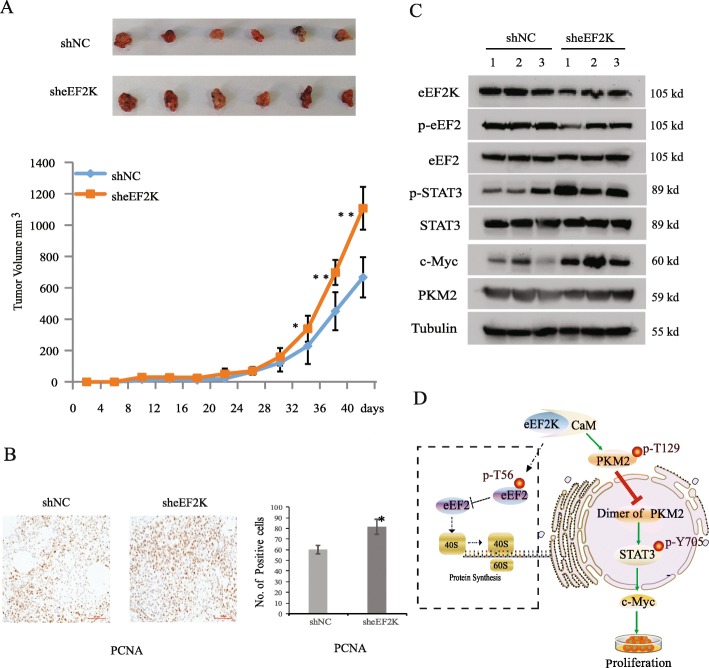
eEF2K depletion promotes tumor growth in mice. **a** shNC- and sheEF2K-expressing A549 cells were implanted (s.c.) into right flanks of Balb/c nude mice as described in Methods. Tumor issues were dissected until 40 days after injection and were photographed. Statistical analysis of the tumor volumes in each group was performed using one-way analysis of variance (Student’s t-test). *n* = 6, *p < 0.05, **p < 0.01, vs shNC. **b** Immunohistochemical (IHC) staining of tumor specimens with an anti-PCNA antibody (left). Quantification of PCNA positive cells from tumor specimens (*n* = 3) from shNC and sheEF2K treated mice (right). The mean and standard deviation are plotted. **c** Immunoblotting analyses of tumor specimens were performed with the indicated antibodies. **d** A model of mechanism underlying eEF2K-regulated metabolism and tumor cell proliferation by phosphorylation of PKM2 and regulation of STAT3 phosphorylation and c-Myc expression. The dotted line in figure represents traditional signaling pathway

To determine the role of eEF2K in tumor growth in mice, we subcutaneously injected A549 cells with or without eEF2K depletion in athymic nude mice. As shown in Fig. 7a, eEF2K depletion promoted tumor growth. In addition, A549 cells with eEF2K depletion formed tumors with 200 mm^3^ volume earlier than the cells without eEF2K depletion (Table 1), highlighting the growth advantage conferred by reduced eEF2K expression. IHC analyses of tumor tissues showed that eEF2K depletion increased the expression of proliferating cell nuclear antigen (PCNA), a marker of cell proliferation (Fig. 7b). Immunoblotting analyses of three tissues as random in each group showed that eEF2K depletion increased STAT3 phosphorylation and c-Myc expression in tumor tissues (Fig. 7c), supporting that eEF2K regulates STAT3 phosphorylation and c-Myc expression and subsequent tumor growth.

**Table 1:** Statistics of tumor volumes with 200 mm^3^ after subcutaneously injection of A549 cells

Vaccination day	1	5	9	13	17	21	25
shNC	0/6	1/6	2/6	2/6	3/6	6/6	6/6
sheEF2K	0/6	4/6	4/6	6/6	6/6	6/6	6/6

## Discussion

eEF2K belongs to a small family of protein kinases, which are termed ‘α-kinases’ on the basis of its active-site geometry and is different from typical protein kinase superfamily [2]. eEF2K is activated under conditions of stress, such as energy exhaustion or nutrient deprivation, and mostly exerts cytoprotective effects, therefore help growth of solid tumors [3]. However, eEF2K was recently revealed to impact in opposing ways on cancer development depending on the stage and probably the type of cancer [10, 16, 17, 31], suggesting that it is necessary to learn more about the role of eEF2K in oncology. We showed here that eEF2K functioned as cancer suppressor in lung cancer cells, which inhibited tumorigenesis by blocking cell proliferation independent of its role in protein synthesis.

STAT3 is constitutively activated in diverse cancer cell types and have been demonstrated to be important for the proliferation and survival of cancer cells^− 37^. Our studies showed that eEF2K impaired the activation of STAT3. As we know that frequent STAT3 activation in cancer cells is largely due to the fact that STAT3 is a point of convergence for numerous tyrosine kinases, including PDGFR, EGFR, AKT, SRC and JAK family, etc. [37] However, eEF2K depletion did not affect the activity of these proteins, suggesting eEF2K may regulate the activity of STAT3 via a previously uncharacterized mechanism.

PKM2 is highly expressed in cancer cells and promotes aerobic glycolysis and cell proliferation in vitro and *in vivo* [19, 20]*.* PKM2 exists as both tetrameric and dimeric forms [24]. Increased level of dimeric PKM2, which is less active in metabolic pathway than the tetrameric form, results in a lower rate of glycolysis [38]. While the PKM2 dimer possessed activity of protein kinase, and directly phosphorylates STAT3 independently of JAK and c-Src pathways [24]. We revealed that PKM2, eEF2K and STAT3 formed a complex and that STAT3 phosphorylation was correlated with expression of c-Myc, a well-known STAT3 target gene and a key contributor to the Warburg effect in most cancer types, leads to both increased aerobic glycolysis and proliferation [33, 39]. Importantly, we demonstrated that eEF2K regulated STAT3 phosphorylation in a PKM2-dependent manner. Our mechanistic studies showed that eEF2K phosphorylated PKM2 at T129, leading to reduce dimerization of PKM2 and inhibit PKM2-mediated STAT3 phosphorylation. In addition, eEF2K depletion increased the nuclear translocation of PKM2, which in turn promoted c-Myc expression. c-Myc-dependent expressions of glycolytic gene expression are responsible for enhanced aerobic glycolysis [27], which is reflected by eEF2K depletion-increased glucose consumption and lactate production. Thus, our findings suggest that eEF2K regulates c-Myc expression via STAT3 phosphorylation and nuclear translocation of PKM2, then effects cell proliferation.

In recent years, non-canonical localizations of PKM2 has been extensively studied. PKM2 was found in the nucleus, mitochondria, and extracellular secretion, which related to novel biological functions in tumor progression [19–21, 40, 41]. A number of studies have demonstrated that dimeric PKM2 occurs in nuclei of cancer cells, where it functions as a co-activator of several transcription factors to modulate the expression of target genes, which subsequently contribute to aberrant metabolism and tumor growth under different physiological and pathological circumstances [24, 42, 43]. In addition, PKM2 in nucleus has been suggested as a novel modulator of genomic instability, cancer stemness, and cancer-associated inflammation, etc. [21–23] Therefore, reduction of nuclear PKM2 could be prioritized as a therapeutic strategy in anticancer research. Posttranslational protein modification is the best understood mechanism for regulating subcellular localization of PKM2, such as phosphorylation, acetylation, succinylation, O-GlcNAcylation, and so on [44–47]. Specially, aberrant oncogenic protein kinases phosphorylate PKM2 at tyrosine, serine and threonine residues to modulate its glycolytic and nonglycolytic functions. Furthermore, several reports support that serine/threonine phosphorylation pertains to the nuclear translocation of PKM2. EGFR-stimulated S37 phosphorylation of PKM2 promotes its nuclear translocation to support metabolic reprogramming in cancer cells [48]; Insulin growth factor 1 (IGF1)- activated AKT in the cytosol directly phosphorylates PKM2 at another serine residue (S202) to promote its nuclear translocation [49]; PKM2 phosphorylation at threonine T454 has been shown to promote its nuclear translocation and non-glycolytic function by an oncogenic proviral insertion in the gene for murine lymphomas 2 (PIM2), which is a serine/threonine protein kinase [50]. In our studies, we first demonstrated that PKM2 is a direct substrate for eEF2K, which can be readily phosphorylated at Thr129 by eEF2K in the presence of CaM, albeit less rapidly than eEF2. PKM2^Thr129^ is highly conserved among species, we found that phosphorylation of PKM2^Thr129^ regulates PKM2 activation by blocking dimerization of PKM2, thus, an approach to retain eEF2K could be alternative therapy strategy in intervening formation of dimer PKM2. In fact, eEF2 shouldn’t have been recognized a unique substrate of eEF2K, AMPK and alpha4 were reported to be new substrates of eEF2K before [51], suggesting that eEF2K has a more diverse role in regulating cellular energy and proliferation that involves multiple pathways.

## Conclusion

In summary, our findings constitutes an additional paradigm of eEF2K as a suppressor of carcinogenesis other than a role of cytoprotection in lung cancer cells, and delineate a novel mechanism underlying regulation of proliferation by eEF2K-mediated PKM2 phosphorylation and subsequent dimerization, leading to inhibit STAT3 phosphorylation and c-Myc expression accompanied by collapse of aerobic glycolysis (Fig. 7d). These effects of eEF2k are independent of its role in protein synthesis. Our study discloses the diverse role of eEF2K in cell biology and complexity of eEF2K in cancer development, which lays foundation for the development of new anticancer therapeutic strategies.

## Supplementary information

**Additional file 1: Figure S1.** The phosphorylation level and activity of eEF2K in A549 when cultured with glucose-free or serum-free mediun. **Figure S2.** eEF2K inhibition by A484954 alleviates the effects of gefitinib. **Figure S3.** eEF2K positively regulates the glycolytic enzyme activity of PKM2. **Figure S4.** eEF2K inhibition increases STAT3 phosphorylation in H1299 cells. **Figure S5.** eEF2K affects protein tyrosine kinase activity of PKM2 in the cytoplasm and nucleus extracts. **Figure S6.** Phosphorylation kinetics of known eEF2K substrates.

## Data Availability

The datasets supporting the conclusions of this article are included within the article and its additional files.

## Abbreviations

ADP: Adenosine diphosphate
AKT: Protein kinase B
AMP: Adenosine monophosphate
AMPK: Adenosine 5‘-monophosphate (AMP)-activated protein kinase
ATP: Adenosine triphosphate
CaM: Ca2+/calmodulin
CHX: Cycloheximide
eEF2K: Eukaryotic elongation factor-2 kinase
EGFR: Epidermal growth factor receptor
ERK: Extracellular regulated protein kinases
JAK: Janus Kinase
mTORC1: mammalian target of rapamycin complex 1
MTT: 3-(4,5-dimethyl-2-thiazolyl)-2,5-diphenyl-2-H-tetrazolium bromide
PCNA: Proliferating Cell Nuclear Antigen
PDGFR: Platelet-derived growth factor receptor
PEP: Phosphoenolpyruvate
PI3K: Phosphatidylinositol 3 kinase
PKM: The M Isoform of Pyruvate kinase
STAT3: Signal Transducers and Activators of Transcription 3
WT: Wild-type
