# Supplementary material for: The eEF2 kinase-induced STAT3 inactivation inhibits lung cancer cell proliferation by phosphorylation of PKM2
Source: Cell Commun Signal. 2020 Feb 13;18:25. doi: 10.1186/s12964-020-0528-y (PMC7020344; doi:10.1186/s12964-020-0528-y)
Supplement: Supplementary file 1 — Additional file 1: Figure S1. The phosphorylation level and activity of eEF2K in A549 when cultured with glucose-free or serum-free mediun. Figure S2. eEF2K inhibition by A484954 alleviates the effects of gefitinib. Figure S3. eEF2K positively regulates the glycolytic enzyme activity of PKM2. Figure S4. eEF2K inhibition increases STAT3 phosphorylation in H1299 cells. Figure S5. eEF2K affects protein tyrosine kinase activity of PKM2 in the cytoplasm and nucleus extracts. Figure S6. Phosphorylation kinetics of known eEF2K substrates. [file 12964_2020_528_MOESM1_ESM.zip › Additional file 1.docx]

Additional file 1

**Fig.S1: The phosphorylation level and activity of eEF2K in A549 when cultured with glucose-free or serum-free mediun.**

Cells were cultured with 6-well plates in complete medium for 12 h, then maintained in glucose-free or serum-free medium. Immunoblotting analyses were performed with the indicated antibodies.

**Fig.S2: eEF2K inhibition by A484954 alleviates the effects of gefitinib.**

(A) A549 cells were treated with A484954. Immunoblotting analyses were performed with the indicated antibodies. (B) A549 with or without A484954 were treated with Gefitinib at the indicated concentrations The cell viability was detected by MTT assay. The histogram shows the percentages of the viable cells. Data are expressed as mean of the percentages of three experiments. **p<0.01, vs DMSO.

**Fig. S3: eEF2K positively regulates the glycolytic enzyme activity of PKM2**

(A)Glycolytic enzyme activity of PKM2 was detected with PKM2 activity assay kit (Biovision K709-100 ). Briefly, cell lysates of sh/WT-eef2k A549 cells were added to a 96-well plate to a final concentration of 0.3 μg/ml. The absorbance at λ=570 nm is detected by Epoch2 microplate reader (Bio-TEK) at 1 min (Δ570 nm). Taking the absorbance values of 15 min (ΔB570 nm) and 5 min (ΔA570 nm) to calculate the difference Δ570 nm, the PKM2 activity inhibition rate =（ΔB570 nm -ΔA570 nm）/ ΔA570 nm. (B) Standard curve for pyruvate measurement performed according to the manufacturer’s protocol. Results are mean ± sd. **P < 0.01 vs Ctrl.

**Fig. S4: eEF2K inhibition** **increases STAT3 phosphorylation in H1299 cells**

H1299 cells with or without eEF2K depletion were cultured for the indicated periods of time. Immunoblotting analyses were performed with the indicated antibodies.

**Fig.S5 eEF2K affects protein tyrosine kinase activity of PKM2 in the cytoplasm and nucleus extracts.**

Cytoplasm (A) and nuclear (B) were separated by Cytoplasm / Nuclear Isolation Kit (BestBio:BB-3602-1). The nuclear or cytoplasmic extracts of sh/WT-eef2k expression of A549 cells were incubated with 10 μg/ml of recombinant human GST-STAT3 (Abcam: ab43618) under various conditions (with or without 5mM PEP) with kinase buffer (50 mM Tris-HCl, pH = 7.5, 100 mM KCl, 50 mM MgCl_2_,1 mM Na_3_VO_4_, 1 mM PMSF, and 1 mM DTT) in 100 μl volume for 1 hours at 37℃. The reaction mixtures were then subjected to 10% SDS-PAGE analyses. Tubulin and H3 were used as loading control for the cytoplasm and nuclear components, respectively.

**Fig.S6: Phosphorylation kinetics of known eEF2K substrates**

(A) An *in vitro* phosphorylation assay was performed by incubation of the indicated amount of eEF2K, 8 μM ATP, CaM, and PKM2 in the presence or absence of 5 μM JAN-384. (B, C) An *in vitro* phosphorylation assay was performed by incubation of 1 μg eEF2K, 8 μM ATP, and calmodulin in the presence of purified eEF2 or PKM2 (B). Quantification of data were performed (C).
